# sTREM2 is associated with attenuated tau aggregate accumulation in the presence of amyloid-β pathology

**DOI:** 10.1093/braincomms/fcad286

**Published:** 2023-10-24

**Authors:** Fardin Nabizadeh

**Affiliations:** School of Medicine, Iran University of Medical Sciences, Tehran, 1449614535, Iran

**Keywords:** TREM2, Alzheimer’s disease, microglia, amyloid β, tau

## Abstract

Triggering Receptor Expressed on Myeloid Cell 2 (TREM2) plays a crucial role in the transition of microglia from a state of homeostasis to a state associated with the disease. Mutations in TREM2 are strongly linked with a higher risk of developing neurodegenerative diseases, including Alzheimer’s disease. There have been contradictory findings regarding the potential detrimental or protective effects of microglial activation and TREM2-related microglial responses in Alzheimer’s disease. Although previous studies reported increased CSF soluble TREM2 (sTREM2) in different clinical stages of Alzheimer’s disease, the exact association between Alzheimer’s disease hallmarks such as amyloid-beta and tau pathology remains unclear. In the present study, I aimed to investigate the association between TREM2-related microglial responses and tau accumulation in the presence and absence of amyloid-beta pathology in order to give a better view of the role of microglial activation in Alzheimer’s disease development. Imaging data of 178 non-demented participants including 107 amyloid-beta-negative participants, 71 amyloid-beta-positive were recruited from Alzheimer’s disease Neuroimaging Initiative. The CSF sTREM2 was used as an *in vivo* indicator of microglial responses associated with TREM2. Furthermore, I used longitudinal tau-PET and resting-state functional MRI connectomes in order to investigate the association of TREM2-related microglial activation and tau spreading through functional connections. A higher level of sTREM2 was associated with slower tau aggregate accumulation in non-demented amyloid-beta-positive. Furthermore, measuring the tau spreading through inter-connected regions using functional MRI connectomes confirms that the TREM2-related microglial activity might be a protective factor against tau pathology in brain tissue. These findings demonstrate that in individuals with initial amyloid-beta abnormalities, TREM2-related microglial activation is linked to reduced regional accumulation of tau aggregates and also, spreading across inter-connected brain regions, as evaluated through functional MRI connectomes during the early stages of Alzheimer’s disease.

## Teaser

Nabizadeh *et al*. reported that the soluble Triggering Receptor Expressed on Myeloid Cell 2 in CSF is associated with slower tau aggregate accumulation. Also, it might be a protective factor against tau spreading through inter-connected regions in brain tissue.

## Graphical Abstract

**Graphical Abstract:**
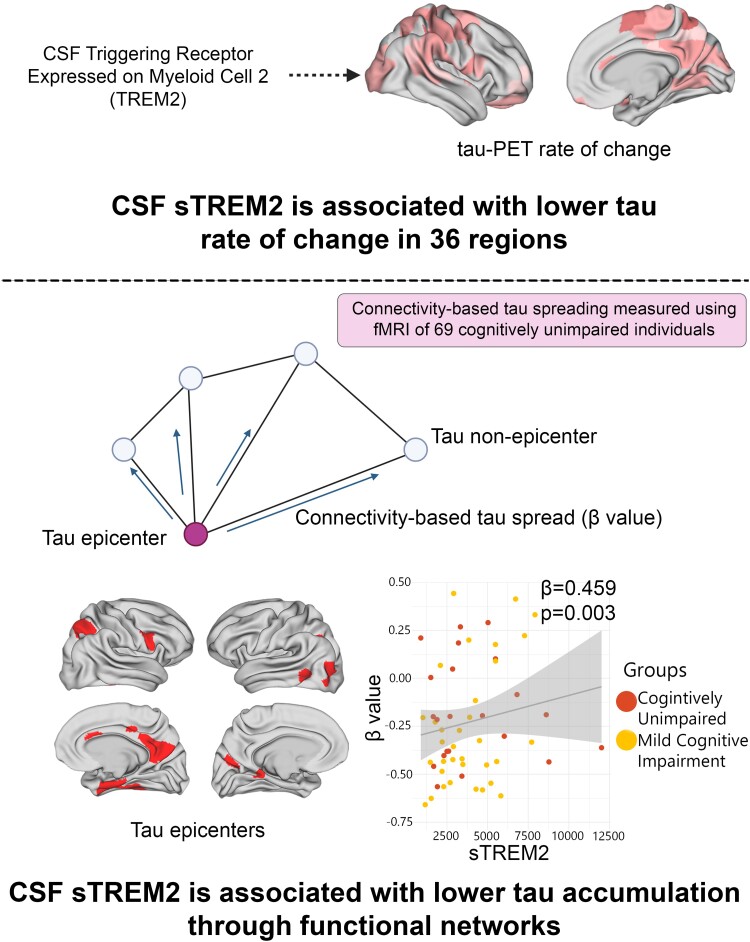

## Introduction

Alzheimer's disease (AD) is an age-related neurodegenerative disorder that is the primary form of dementia. The disease is characterized by the accumulation of extracellular beta-amyloid (Aβ) aggregates, intracellular tau neurofibrillary tangles (NFT) and neuronal cell death, leading to cognitive decline.^1^ Previous imaging and biomarkers studies lead to the amyloid cascade hypothesis, which revealed that the accumulation of Aβ triggers the sequential formation and spreading of hyperphosphorylated tau aggregates, ultimately leading to neurodegeneration, metabolic impairment and eventual dementia.^2,3^ The greatest risk factors of Alzheimer's disease are age, female gender and the presence of the apolipoprotein E (APOE) ɛ4 allele.^4^ The higher incidence of Alzheimer's disease in females is commonly attributed to their longer lifespan in comparison to males.^5^

The APOE is the major carrier of cholesterol and various lipid species within the central nervous system.^6^ Despite conducting numerous genome-wide association studies (GWAS) and GWAS meta-analyses, the ɛ4 allele of the APOE gene, which was first identified as a risk factor for sporadic Alzheimer's disease in 1993, remains the most substantial genetic susceptibility for this disease that can increase the risk of developing AD 2- to 3-fold with having at least APOE ɛ4 allele.^7^ The exact mechanism of how APOE ɛ4 increases the susceptibility to Alzheimer's disease remains unclear, but it is possible that the diminished capacity of neurons to metabolize lipids may be a significant contributing factor.

It has become evident that the amyloid cascade hypothesis is inadequate in providing a complete explanation for the accumulation of aggregated Aβ and tau in Alzheimer's disease.^8^ In fact, the development of Alzheimer's disease might be a consequence of brain cell homeostasis disruption. Microglia, the primary immune cells of the brain, have emerged as the primary responders to brain damage and have been extensively studied in relation to Alzheimer's disease.^9^ The activation of microglia may have an important role in the modulating of the initial events in the amyloid cascade.^10^ Triggering Receptor Expressed on Myeloid Cell 2 (TREM2) plays a crucial role in the transition of microglia from a state of homeostasis to a state associated with disease.^11,12^ It is also recognized as a reliable *in vivo* indicator of microglial activation in Alzheimer's disease.^13^ TREM2 plays a key role in the functions of microglia, such as phagocytosis, the release of cytokines, lipid sensing and the proliferation and migration of microglia.^14,15^ Mutations in TREM2 are strongly linked with an higher risk of developing neurodegenerative diseases, including AD,^16^ frontotemporal dementia (FTD),^17^ Parkinson’s disease (PD) and amyotrophic lateral sclerosis (ALS).^18^

Soluble TREM2 (sTREM2) is found in the conditioned media of cultured cells as well as in biological fluids such as plasma and cerebrospinal fluid (CSF).^19^ The shedding process is mediated by ADAM10 and 17, which occurs at the C-terminal of histidine 157.^20^ Homozygous mutations that lead to FTD-like syndrome, such as p.T66M, retain misfolded TREM2 in the endoplasmic reticulum, which prevents its maturation and cleavage on the plasma membrane. Consequently, individuals carrying these mutations have undetectable levels of sTREM2 in their CSF and blood.^21^ Given that the selective expression of TREM2 in microglia within the CNS is linked to both AD and neurodegeneration, let us hypothesize that sTREM2 in CSF could serve as an indicator of microglial function and its response to Aβ and tau pathologies, as well as neurodegeneration. To be more precise, the quantity of sTREM2 may indicate the level of signalling-competent TREM2 present on the surface of activated microglia. This hypothesis is supported by animal studies.^21^

There have been contradictory findings regarding the potential detrimental or protective effects of microglial activation and TREM2-related microglial responses in Alzheimer's disease.^22^ Notably, recent *in vitro* studies have indicated that activated microglia may contribute to tau hyperphosphorylation and spread, as well as release tau seeds that promote tau aggregation.^23^ Likewise, investigations conducted on patients with sporadic Alzheimer's disease have found an association between a TREM2-related microglial reaction and soluble p-tau, but not with Aβ levels.^24^ In addition, there is evidence suggesting that a microglial response related to TREM2 may facilitate the development of aggregated tau pathology in Alzheimer's disease, as measured by tau-positron emission tomography (PET).^10,25^ A recent study post-mortem study revealed that microglial activation has a mediating effect of 33% on the association between Aβ and tau in brain tissue. This indicates that microglial activation could be associated with tau hyperphosphorylation and potentially play a role in the development of tau pathology in Alzheimer's disease.^26^ Conversely, a higher TREM2-related microglial response has been associated with reduced cognitive decline, amyloid accumulation and neurodegeneration in individuals with symptomatic sporadic and autosomal dominant Alzheimer's disease.^27,28^

It is crucial for clinical trials aimed at targeting microglial activation as a disease-modifying approach to understand the potential protective or detrimental role of microglial activation, as well as the effect of microglial on the progression of Alzheimer's disease considering the stage of the disease.

In the present study, I aimed to investigate the association between TREM2-related microglial responses and tau accumulation in the presence and absence of Aβ pathology in order to give a better view of the role of TREM2-related microglial activation in Alzheimer's disease development. The CSF sTREM2 was used as an *in vivo* indicator of microglial responses associated with TREM2. Furthermore, I used longitudinal tau-PET and resting-state functional magnetic resonance imaging (fMRI) connectomes in order to investigate the association of TREM2-related microglial activation and tau spreading through functional connections.^29^

## Methods

### Participants

The Alzheimer’s disease Neuroimaging Initiative (ADNI) study was established in 2003 as a public–private partnership with the primary objective of evaluating the possibility of combining serial MRI, PET, other biological markers and clinical and neuropsychological assessments to monitor the progression of mild cognitive impairment (MCI) and early Alzheimer’s disease. For current and up-to-date information on this study, please visit http://adni.loni.usc.edu. This current investigation utilized data from ADNI3 (NCT02854033), which was approved by all appropriate ethical boards linked to ADNI (refer to the Reporting Summary). All participants provided informed consent, and they received compensation for their participation in the study. From the ADNI database, 184 individuals with tau-PET were chosen for the study based on the availability of longitudinal tau-PET, Aβ-PET (florbetaben or florbetapir) and sTREM2. Only 178 participants were ultimately included in the study, as the remaining six were diagnosed with AD dementia and were excluded due to the insufficient sample size. ADNI assessed the clinical status of participants and identified those with a Mini-Mental State Exam (MMSE) score > 24, a Clinical Dementia Rating (CDR) score of 0 and no depression as cognitively unimpaired (CU) participants, while those with MCI had an MMSE score > 24, a CDR of 0.5, objective memory impairment based on education-adjusted Wechsler Memory Scale II and preserved activities of daily living. All methods were performed in accordance with the relevant guidelines and regulations.

### Image acquisition and processing

Further information on the PET and MRI image acquisition procedures in the ADNI cohort can be obtained from an external source (http://adni.loni.usc.edu/methods/documents/). In brief, 3D T_1_-weighted MPRAGE sequences with 1 mm isotropic voxel size were used to acquire structural MRI data on 3 T scanners. PET data were obtained using 18F-labeled tracers at standardized time intervals post-injection. Images for tau-PET were acquired using flortaucipir 75–105 minutes post-injection. For Aβ-PET, florbetapir (image acquisition 50–70 minutes post-injection) and florbetaben (image acquisition 90–110 minutes post-injection starting in ADNI3) were used. All image processing was conducted locally, and the images were realigned, averaged, resliced to 1.5 mm^3^ and smoothed to a resolution of 8 mm^3^ Full Width Half Maximum (FWHM). The closest structural T_1_-weighted MRI image registered each PET image. The inferior cerebellum and whole cerebellum were used as the reference regions for flortaucipir and florbetapir/florbetaben, respectively, to create standardized uptake value ratio (SUVR) images. ANTs version 2.3.1 was utilized to register each T_1_-weighted image to the MNI template space, and these registration parameters were used to register SUVR PET images to the MNI space for further analyses. The ADNI PET core established thresholds for Aβ positivity, with a cut-off of 1.11 SUVR for florbetapir and 1.08 SUVR for florbetaben in a global composite cortical region referenced to the whole cerebellum.^30^ To enable an analysis of all participants despite using two Aβ tracers, global Aβ SUVR was transformed into the Centiloid scale.^31^

### CSF assessment

To evaluate sTREM2, an Enzyme-linked Immunosorbent Assay (ELISA) approach was utilized, which has been described previously.^14,32^ The sTREM2 data are available in the ADNI database in the ADNI_HAASS_WASHU_LAB.csv file under the variable ‘MSD_STREM2CORRECTED’. Additional information on the methods used can be found online at https://ida.loni.usc.edu. The Luminex platform was used to obtain CSF samples, and the levels of p-tau were measured using Luminex’s micro-bead-based multiplex immunoassay. Additional information regarding the collection of CSF specimens and analytical measurement can be found on the ADNI website (http://adni.loni.usc.edu/methods/documents/).

### Regional measures and rate of change tau- and Aβ-PET

PET images, including Aβ and tau, were divided into 200 regions corresponding to the nilearn.datasets.fetch_atlas_schaefer_2018.^33^ Average SUVR was extracted from these regions, covering the entire neocortex. Grey matter masks were used to mask each region to ensure that the final values were not affected by binding from white matter or CSF. To determine the rate of change in tau-PET over time, linear mixed-effect models were used, with tau-PET SUVR as the dependent variable and time as the independent variable. Participants had between two and four tau-PET scans. The slope of each participant’s model represented the rate of tau aggregate accumulation, which was calculated as the change in tau-PET SUVR per year.

### Defining tau-PET epicentres

Tau-PET epicentres were identified at the individual level using baseline tau-PET data. These epicentres represented regions with the highest probability of being abnormal tau aggregates using Gaussian mixture modelling (GMM). Due to the skewed distribution of tau-PET signal across participants and brain regions, GMM was used to separate target binding from non-specific binding. Two-distribution GMM was fitted on each brain region to separate both signals and extract the probability of falling on the right-most distribution, which reflected an abnormal tau-PET signal. The GMM probability represented the probabilistic measure of tau positivity without using a priori thresholds.^34,35^ The GMMs were fitted on the ADNI sample with longitudinal tau-PET. A few parcels in the somatomotor regions with low tau-PET SUVR were excluded from the epicentre selection. The GMM probabilistic value was multiplied with the tau-PET SUVR to obtain an SUVR score that was cleaned from unspecific signals. The top 10 regions with the highest probability of SUVR weighted by GMM were selected to define tau-PET epicentres at the individual level for further connectivity-based analyses.^36^

### Template and analyses of functional connectivity

In order to investigate whether tau-PET accumulation is associated with functional brain architecture, a template functional connectivity matrix was derived from a group of 69 cognitively unimpaired individuals from the ADNI cohort who were Aβ-negative and had low tau-PET binding. The template matrix was generated using various processing steps, including realignment, co-registration, detrending, band-pass filtering and regression of nuisance covariates. A scrubbing technique was also applied to remove frames with excessive motion, and only participants with <30% of data that needed to be censored were retained. Functional connectivity matrices were created using the Schaefer atlas of 200 parcels, and Fisher-*z* correlations were used to assess subject-specific functional connectivity matrices. The individual matrices were averaged and thresholded at 30% density to obtain an average functional connectivity matrix, which was then converted to a distance-based connectivity matrix. To investigate the relationship between functional connectivity and tau aggregate accumulation, the distance-based functional connectivity of brain regions to the tau epicentres was calculated. The rate of change in tau-PET in each remaining brain region (*n* = 190) was correlated with its connectivity-based distance to the tau epicentres at both the group and individual levels. The strength of the association between tau-PET accumulation and connectivity to tau epicentres across the whole brain was represented as a standardized *β*-value for each participant. Negative *β*-values were expected, indicating that greater tau-PET change was associated with stronger connectivity (represented as smaller values). Notably, adjusting for Euclidean distance between regions of interest (ROIs) or using a functional connectivity matrix based on partial correlations as a template did not alter the results of this approach.^36^

### Statistical analysis

It is important to note that due to the small sample size in ADNI, only analyses with higher than 80% statistical power were performed (effect size = 0.3 and *α* = 0.05). The necessary sample size estimates for each analysis in ADNI were calculated using the R package pwr v1.3-0 and a previous study used a larger sample size.^37^ All analyses were conducted using R version 4.0.5, and the main packages used included stats v4.0.5, lme4 v1.1-30 for linear mixed-effect models with random intercept and random slope, mediation v4.5.0 for mediation analyses and ggplot2 v3.3.6 for creating plots. Brain renderings were created using the Connectome WorkBench software v.1.5.0. The Kolmogorov–Smirnov test was performed to check the normality of the data, and non-parametric variables were log-transformed to be appropriate for parametric assumptions.

To investigate the main factors related to the accumulation of tau aggregates (measured as tau-PET rate of change), several linear regression models were applied to each of the 200 brain parcels. The dependent variable in each model was the tau-PET rate of change in each parcel, with age and sex as covariates. The models included progressively more dependent variables, starting with regional Aβ-PET SUVR alone, then sTREM2 alone, followed by regional Aβ-PET SUVR and sTREM2 and finally regional Aβ-PET SUVR, sTREM2 and baseline regional tau-PET SUVR. Results were reported for regions where coefficients of regional Aβ-PET SUVR or sTREM2 were considered significant after false discovery rate (FDR) correction at a *P*-value < 0.05. Multicollinearity between Aβ-PET SUVR and sTREM2 was also checked to ensure that there was no redundancy in the models. The results of the linear models were used to test the mediating effect of sTREM2 on regional Aβ-PET SUVR and regional tau-PET rate of change region-wise. Mediation analyses were conducted using the R package mediation version 4.5.0, with all paths of the mediation model controlled for age and sex. The significance of the mediation effect was determined using 1000 bootstrapping iterations.

Linear models were applied to each participant, evaluating the relationship between connectivity to tau epicentres and the tau-PET rate of change. This resulted in a *β*-value that was then correlated with sTREM2 concentrations, taking into account age, sex and global Aβ-PET.

The study concentrated on the relationships between sTREM2 measures and cognitive decline. To test the mediating effect of (i) the *β*-value linking connectivity with tau aggregate accumulation throughout the brain and (ii) the tau-PET rate of change on CSF sTREM2 and cognitive decline, the researchers performed mediation models as described earlier, controlling for age and sex for all paths in these models. Cognitive decline was measured either as the slope of MMSE.

### Ethical approval

Since the data in this paper were obtained from the ADNI database (adni.loni.usc.edu), it does not include any research involving human or animal subjects.

The STROBE checklist was followed in this observational study.

## Results

### Study design and participants

In total, 178 participants including 47 CU Aβ-negative participants, 27 Aβ-positive CU, 60 Aβ-negative patients with MCI and 44 Aβ-positive MCI patients entered the study. The Aβ-positive CU and MCI participants were combined into a single group named ‘non-demented participants’, with the aim to study the early stages of Alzheimer's disease. In addition, all the main analyses were carried out for each group separately. All PET data were parcellated in 200 cortical ROIs using the Schaefer brain atlas.^33^ When conducting analyses across all 200 brain regions, only the findings that remained statistically significant after multiple comparisons from the FDR were reported. The sTREM2 concentrations were measured in the CSF. To quantify the accumulation of tau protein aggregates, the rate of change in tau-PET retention over time (SUVR/year) was evaluated for each brain region independently, using linear mixed-effect models. Additionally, cognitive decline was assessed by measuring the rate of change in cognitive scores (MMSE) per year, using linear mixed-effect models. The baseline demographic and clinical characteristics of the participants were detailed in Table 1. The investigated time points of participants are represented in Supplementary 1.

**Table 1:** Demographic and clinical characteristics

Variable	Aβ-negative non-demented (*n* = 107)	Aβ-positive non-demented (*n* = 71)
Age (years)	69.7 ± 5.7	72.4 ± 6.2
Female (%)	51 (47%)	36 (51%)
Education (years)	16.7 ± 2.5	15.7 ± 2.9
APOEɛ4 carriers (%)	31 (29%)	35 (49%)
MMSE	28.9 ± 1.1	28.3 ± 1.6
CSF sTREM2 (pg/ml)	3697.6 ± 2039.5	3916.9 ± 2301.3
tau-PET follow-up time (years)	3.0 ± 1.6	2.3 ± 1.2
MCI (%)	60 (56%)	44 (62%)

Data are presented as mean ± standard deviation unless specified otherwise.

APOEɛ4, apolipoprotein E genotype (carrying at least one ɛ4 allele); CSF sTREM2, cerebrospinal fluid soluble Triggering Receptor Expressed on Myeloid Cell 2; MMSE, Mini-Mental State Exam; PET, positron emission tomography; MCI, mild cognitive impairment.

### Increased sTREM2 is the main modifier of tau-PET accumulation rates in the presence of Aβ pathology

**Figure 1:**
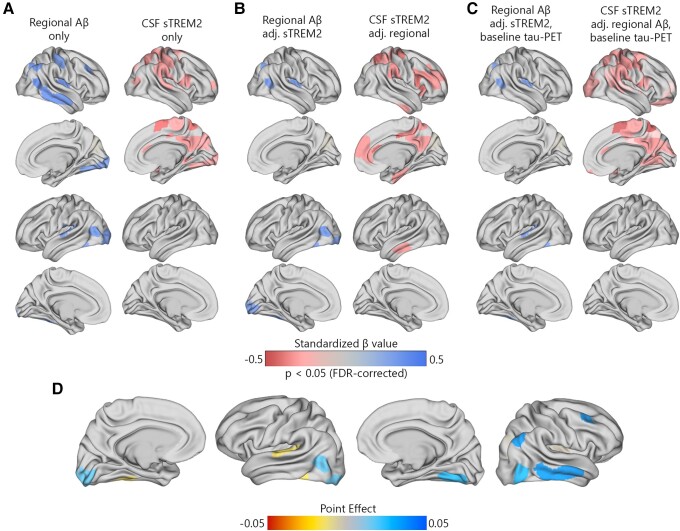
**Regional Aβ-PET and CSF sTREM2 associations with regional tau-PET rate of change in Aβ-positive non-demented participants.** (**A**) The standardized beta coefficient of local Aβ-PET in brain regions where the regional Aβ-PET SUVR (left column) is associated with the regional tau-PET rate of change, while adjusted for the effects of age and sex. The right columns present a similar model, but using CSF sTREM2 as a predictor instead of Aβ-PET. (**B**) The standardized beta coefficient of local Aβ-PET (left column) and CSF sTREM2 in regions associated with the regional tau-PET rate of change while adjusted for Aβ-PET, CSF sTREM2, age and sex. (**C**) Similar visualization to (**B**) while additionally adjusted for regional baseline tau-PET SUVR. (**D**) The mediating effect of CSF sTREM2 on local Aβ-related accumulation of local tau aggregates. The mediation models were performed region-wise, and the point effects are visualized on the brains.

Primarily, I used linear regression models to investigate the relationship between the concentration of sTREM2 and the level of local Aβ aggregates with local increases in insoluble tau aggregates in 200 brain regions during the early stages of AD. I aimed to determine which factor had the strongest association with these increases over time. In non-demented Aβ-positive participants, there was a notable significant correlation between regional Aβ and the accumulation of tau aggregates over time, particularly in the temporo-parietal regions (Fig. 1A). Following correction for multiple comparisons, the standardized *β*-value of Aβ was calculated across 17 regions, with an average value of 0.23 and a range spanning from 0.19 to 0.46. The average *P*-values observed in these regions were 0.029 (Supplementary 2). The association between levels of sTREM2 and the accumulation of tau aggregates displayed a negative correlation in non-demented Aβ-positive participants. This correlation was observed in a greater number of brain regions (*n* = 36), compared to the correlation observed between regional Aβ and tau aggregates. Furthermore, the observed standardized *β*-value was significantly higher (Fig. 1A, average standardized *β*-value: −0.39, range −0.23 to −0.48, average *P*-values 0.028).

When using regional Aβ and sTREM2 concurrently as predictors (Fig. 1B), the extensive effect of sTREM2 on the accumulation rate of tau remained largely consistent. However, after adjusting for sTREM2, the independent effect of regional Aβ on tau accumulation was almost negligible. Moreover, all key regions that exhibited the strongest association between sTREM2 and the accumulation of tau aggregates over time remained statistically significant when adjusted for regional Aβ and tau aggregates (Fig. 1C, average standardized *β*-value: −0.32, range −0.22 to −0.44, average *P*-values = 0.031).

Considering the observed interplay between local Aβ and sTREM2 on the increased tau-PET rate of change, a formal analysis was performed to assess the mediating role of sTREM2 in the Aβ-related rate of tau aggregate accumulation at the regional level. The point effect of sTREM2 on the regions that remained significant after multiple comparisons (*n* = 17) is presented in Fig. 1D. The findings suggest that increased levels of sTREM2 do not mediate the impact of local Aβ aggregates on the accumulation rate of insoluble tau aggregates over time (Fig. 1D).

### sTREM2 levels relate to connectivity-based accumulation of tau aggregates in the presence of Aβ pathology

**Figure 2:**
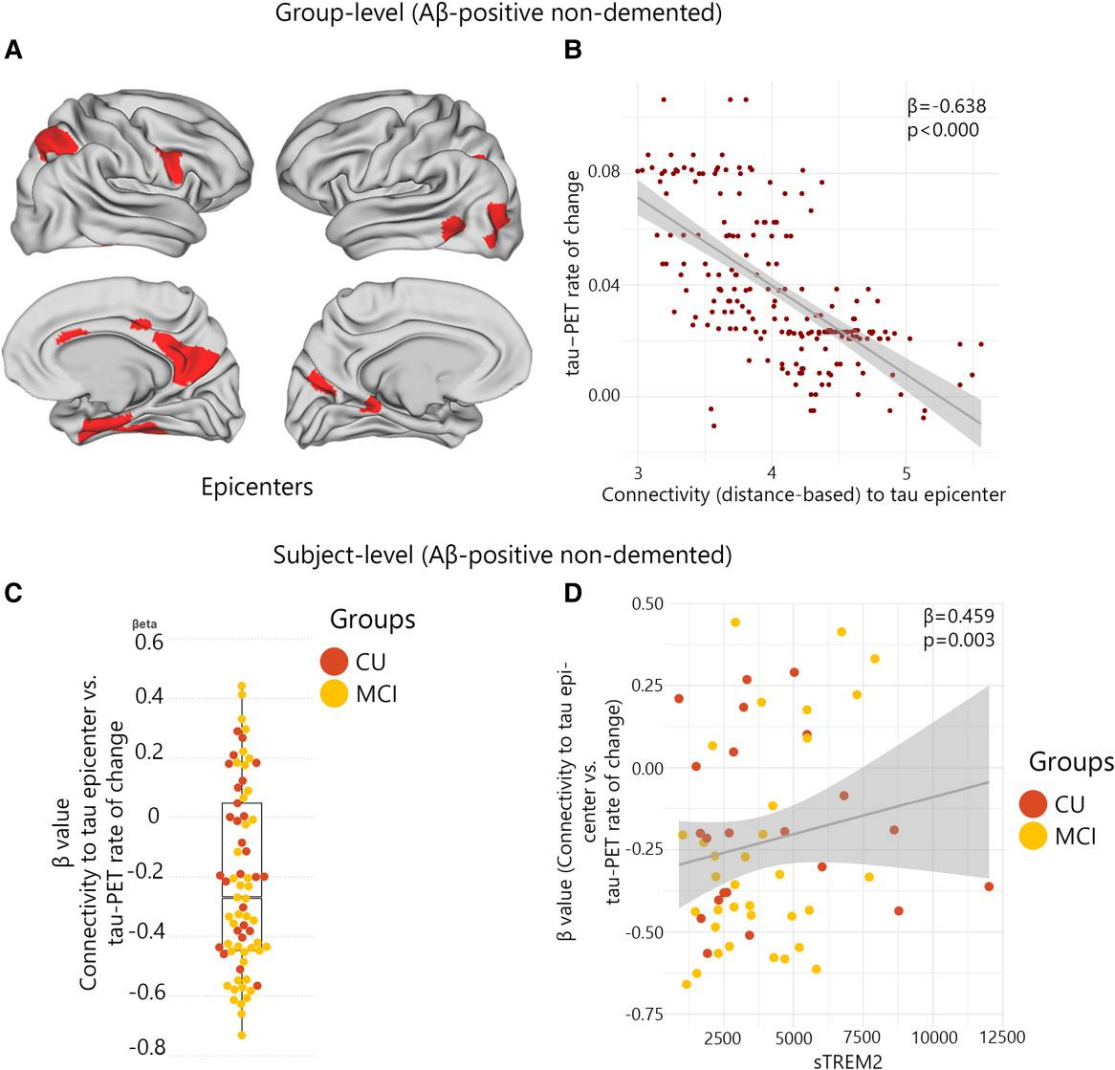
**Group and subject level connectivity-based associations of the tau-PET rate of change over time and CSF sTREM2 in Aβ-positive non-demented participants.** (**A and B**) Results of the group-level analysis that investigated the association between connectivity to the tau epicentres, as visualized on glass brains, and tau-PET rate of change across the entire brain. Each dot represents a distinct brain region. Regions that are more functionally closer to the epicentres exhibit a higher rate of tau-PET accumulation. (**C**) At the individual level, the same approach as (**A and B**) was used. The box plot displays the individual *β*-value resulting from the correlation analysis between the rate of change in tau-PET and the connectivity-based distance to epicentres across all brain regions. (**D**) Scatter plot demonstrating the correlation between CSF sTREM2 and the *β*-values of connectivity between epicentres and tau-PET rate of change adjusting for age, sex and global Aβ-PET. Each dot on the graph represents a unique individual. Our findings reveal a positive association, indicating that higher CSF sTREM2 levels are correlated to a decrease in tau-PET change in regions that are more functionally connected to epicentres. CN, control normal; MCI, mild cognitive impairment.

The functional architecture of the brain plays a pivotal role in regional tau accumulation. Thus, I aimed to investigate whether the concentration of sTREM2 affects the connectivity-based tau accumulation and its spread throughout the functional connections brain. I identified the top 10 regions with the highest tau-PET SUVR probability from GMM at baseline, which is defined as participant-specific tau-PET epicentres. I subsequently performed a correlation analysis between the accumulation of tau aggregates in the remaining 190 regions and their functional connectivity strength to the epicentres. The model was first implemented at the group level, which revealed a greater rate of tau aggregate accumulation in regions exhibiting a stronger connection to the epicentres, as indicated by shorter distance-based connectivity, non-demented Aβ-positive participants (Fig. 2A). The result was the same in non-demented Aβ-negative participants. Following the initial group-level analysis, the model was applied at the individual level by defining tau epicentres for each participant. My analysis revealed a greater rate of tau aggregate accumulation in regions with the strongest connectivity to the tau epicentres using baseline tau-PET. Negative *β*-values (i.e. demonstrating the association between connectivity to the epicentres and rate of tau aggregate accumulation in all non-epicentre ROIs) evidenced these associations (Fig. 2B).

Next, based on the observed association between sTREM2 and the accumulation of tau aggregates, I hypothesize that the higher level of sTREM2 would be related to a weaker association between connectivity to the epicentres and the accumulation rates of tau aggregates across brain regions (Fig. 2C). My analysis reveals that non-demented Aβ-positive participants with higher levels of sTREM2 showed reduced association between connectivity to the epicentres and rate of tau aggregate accumulation in non-epicentre ROIs (*β*-value) after adjusting for global Aβ, age and sex (Fig. 2D). This association remained significant after adjusting for baseline tau-PET (*β* = 0.388, *P*-value = 0.011). Conversely, no significant correlation was found in non-demented Aβ-negative participants that indicates that this effect can only be seen in non-demented Aβ-positive participants who tend to show a stronger association between connectivity to epicentre and tau-PET rate of change (more negative *β*-values, Fig. 2D).

### Connectivity-based tau-PET change mediates the associations between sTREM2 and cognitive decline in the presence of Aβ pathology

**Figure 3:**
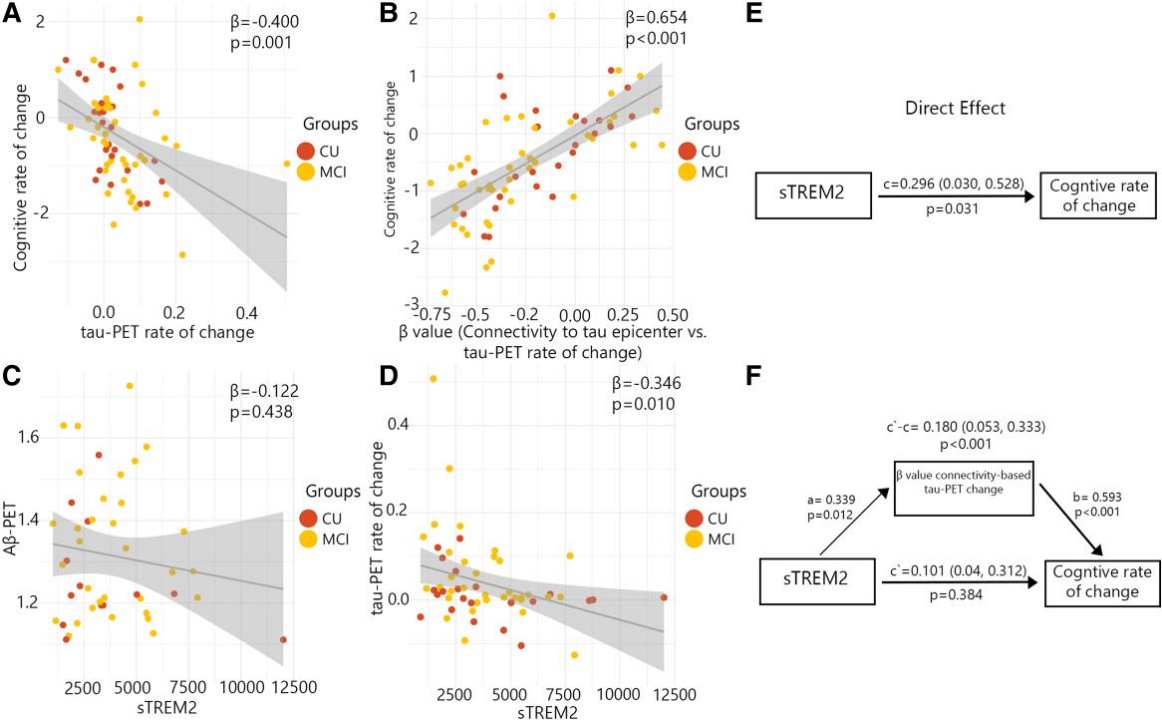
***β*-Values of connectivity between epicentres and tau-PET rate of change mediate associations between CSF sTREM2 and cognitive decline in Aβ-positive non-demented participants.** (**A–D**) Scatter plots of associations relevant to subsequent mediation analyses, beta coefficients from linear regressions adjusting for age and sex. (**A**) Association between global tau-PET rate of change and cognitive rate of change. (**B**) Association between *β*-values of connectivity between epicentres and tau-PET rate of change and cognitive rate of change. (**C**) Association between global Aβ-PET and CSF sTREM2. (**D**) Association between global tau-PET rate of change and CSF sTREM2. (**E**) The direct effect of CSF sTREM2 on cognitive decline is shown. (**F**) Mediation analysis is shown with *β*-value based on connectivity and tau-PET change.

After determining the association between sTREM2 and the regional accumulation of tau aggregates and connectivity-based tau accumulation, I proceeded to investigate how these multiple measures of tau pathology and sTREM2 related to cognitive decline early AD (non-demented Aβ-positive participants). The various measures of tau pathology including global tau-PET rate of change, and the *β*-value of connectivity-based tau-PET rate of change, were all correlated with cognitive decline measured by annual changes of MMSE over a period of time (Fig. 3A and B).

Subsequently, I investigated whether measures associated with the accumulation rate of tau aggregates mediated the relationship between sTREM2 concentrations and cognitive decline. My analysis revealed that the strength (*β*-value) of the association between the rate of tau aggregate accumulation and functional connectivity to tau epicentres was capable of mediating the association between sTREM2 levels and cognitive decline (*P*-values ≤ 0.001, c′–c = 0.180; Fig. 3F). This finding indicates a partial mediating effect of more rapid connectivity-based tau aggregate accumulation. I repeated the analysis adjusting for the effect of CSF p-tau. The result remained significant (*P*-values ≤ 0.001, c′–c = 0.172). However, the accumulation rate of global tau aggregates did not mediate the association between sTREM2 concentrations and the rate of cognitive decline.

In an exploratory analysis, I examined the potential association between sTREM2 and the baseline global Aβ, as well as the accumulation rate of global tau aggregates. My findings revealed the absence of any significant association between sTREM2 and baseline global Aβ in non-demented Aβ-positive participants (Fig. 3C). On the other hand, my analysis indicated that individuals with elevated levels of sTREM2 exhibited a lower accumulation rate of tau aggregates (Fig. 3D and E).

### Distinct associations between sTREM2 and accumulation of tau aggregates in healthy individuals

**Figure 4:**
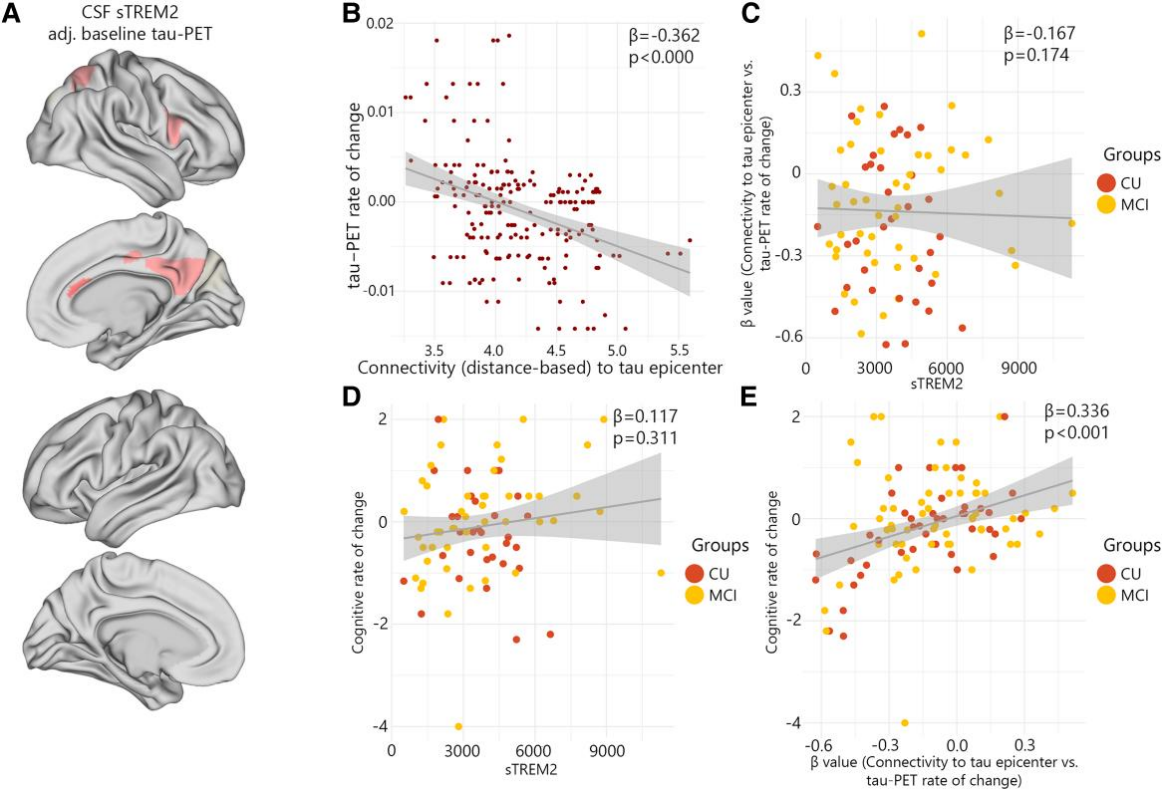
**CSF sTREM2 associations with the regional tau-PET rate of change in Aβ-negative non-demented participants.** (**A**) The standardized beta coefficient of CSF sTREM2 in brain regions where the CSF sTREM2 is associated with the regional tau-PET rate of change, while adjusted for the effects of age, sex and baseline tau-PET. (**B–E**) Scatter plots of associations adjusting for age and sex change in Aβ-negative non-demented participants. (**B**) Group-level analysis of the association between connectivity to the tau epicentres and tau-PET rate of change across the entire brain. (**C**) Association between *β*-values of connectivity between epicentres and tau-PET rate of change and CSF sTREM2 in Aβ-negative non-demented participants. (**D**) Association between CSF sTREM2 and cognitive rate of change. (**E**) Association between *β*-values of connectivity between epicentres and tau-PET rate of change and cognitive rate of change in Aβ-negative non-demented participants.

Previous analysis has exclusively focused on non-demented Aβ-positive participants in order to examine the effects of sTREM2 and connectivity on the rate of change of tau-PET during the early stages of Alzheimer's disease. I repeated the main analyses focusing on non-demented Aβ-negative individuals. My motivation to focus on healthy individuals separately was based on the possible effect of sTREM2 on the accumulation of tau aggregates in the absence of Aβ pathology. Higher levels of sTREM2 in non-demented Aβ-positive participants were linked to a decreased accumulation rate of tau aggregates, adjusting for regional Aβ and baseline tau aggregates. In contrast, sTREM2 levels were found to be significantly associated with the rates of accumulation of tau aggregates in only five regions among healthy individuals while adjusting for baseline levels of tau aggregates (average standardized *β*-value: −0.27, range −0.22 to −0.34, average *P*-values 0.034; Fig. 4A, Supplementary 3).

In Aβ-negative non-demented participants, an expected negative association was observed between the rates of regional accumulation of tau protein aggregates and connectivity to tau epicentres within the brain (Fig. 4B). However, the strength of association (*β*-value) was not correlated with sTREM2 levels (Fig. 4C). Similarly, sTREM2 concentrations did not have a correlation with cognitive decline (Fig. 4D). Rather, the *β*-value of connectivity-based tau-PET rate of change was most associated with cognitive decline in healthy individuals (Fig. 4E).

## Discussion

The present study aimed to assess the TREM2-related microglial responses measured CSF sTREM2 during the Aβ pathological changes. To this end, I classified non-demented participants based on the presence or absence of Aβ pathology that enable us to unravel the effect of Aβ on the association between TREM2-related microglial activity and tau pathology. While there was no association between sTREM2 and Aβ accumulation, a higher level of sTREM2 was associated with slower tau aggregate accumulation in the presence of Aβ pathology. Furthermore, measuring the tau spreading through inter-connected regions using fMRI connectomes confirms that the TREM2-related microglial activity might be a protective factor against tau pathology in brain tissue. However, while the significant association between sTREM2 and longitudinal tau pathology was observed only in non-demented Aβ-positive participants, mediating analysis at the regional level suggests that sTREM2 does not mediate the impact of local Aβ aggregates on the accumulation rate of insoluble tau aggregates over time. Together, these results suggest that microglial responses associated with TREM2 can attenuate tau aggregate accumulation in the presence of Aβ pathology. Previously, Ewers *et al*.^27^ reported lower tau-PET and attenuated Aβ-PET increase suggesting that the higher microglial activation might be protective against subsequent amyloid accumulation. In their study, the results were later confirmed in mouse models. Another study found that TREM2-related microglial responses were associated with reduced neurodegeneration and symptom progression in later disease stages.^13^

My first finding indicated that higher sTREM2 was associated with lower tau aggregate accumulation over time in non-demented Aβ-positive participants that was sustained after adjusting for regional level of Aβ aggregates. Moreover, in contrast to another study that showed that higher sTREM2 mediated the earliest Aβ-related increases in soluble p-tau181 levels, there was no mediation effect for sTREM2 in my study.^38^ These findings support the view that the early formation of Aβ fibrils leads to a TREM2-related microglial activation, which in turn is associated with a slower tau aggregate accumulation, uncoupled from the severity of Aβ deposition. However, there are conflicting findings regarding the role of microglial activation associated with TREM2 on the development of tau pathology. Several preclinical investigations have demonstrated that activated microglia trigger tau phosphorylation in animal models of Alzheimer's disease and other tauopathies.^22,39-41^ Another study on brain tissue samples of Alzheimer's disease patients and mice demonstrated that microglia effectively phagocytose hyperphosphorylated tau seeds, yet are not fully able to neutralize the tau seeding activity.^42^ Instead, microglia release the pathological tau seeds into the extracellular space, leading to a potential cascade of subsequent tau hyperphosphorylation, misfolding and dissemination. However, in line with my findings, other studies indicate that the loss of TREM2 function is associated with a significantly higher risk of Alzheimer's disease and the facilitated Aβ-associated tau seeding in Alzheimer's disease mice affected in the presence of both Aβ and tau pathologies.^43,44^ Further, in symptomatic patients, a higher level of sTREM2 was associated with lower future tau-PET levels.^27^ Various factors, such as the use of different animal models of Alzheimer's disease that mirror different aspects of the Alzheimer's disease pathophysiology or using different markers of tau pathology such as soluble and fibrillar forms of tau, may account for the conflicting results regarding the effect of microglial activation on the development of tau pathology. One of the earliest tau pathology changes in Alzheimer's disease is observed in the CSF p-tau that reflects hyperphosphorylated tau in its soluble form and is closely associated with Aβ, precedes the development of intracellular neurofibrillary tau aggregates.^36,45^ Consequently, further investigations are necessary to comprehensively examine the effect of microglia on hyperphosphorylation and aggregation of p-tau. Also, most of the clinical studies are conducted on well-known cohorts such as ADNI that can be a reason for replicated findings. Furthermore, TREM2-related microglial activation measured by sTREM2 seems to have different natures and associations with Alzheimer's disease biomarkers in each stage of the disease.^24,32,38^

Lee *et al*.^46^ study demonstrated that TREM2 deletion further exacerbated tau aggregate accumulation and spreading only in the presence of Aβ pathology in mice models. This is consistent with my findings that the negative correlation between sTREM2 and the spreading or accumulation of tau aggregates is restricted to individuals with positive Aβ pathology. However, my findings highlight that TREM2-related microglial response plays a crucial role in tau pathology, although the directionality of the effects may be altered by the stage of the disease and other pathophysiological events such as Aβ. It is important to note that microglia exhibit various dynamic states, which respond to a range of physiological and pathological conditions. One of the current limitations is the lack of alternative biomarkers that can accurately reflect the diverse range of microglia states. Proteases of the ADAM family shedding cell-surface full-length TREM2, resulting in the release of sTREM2 into various biological fluids, such as CSF. The influence of sTREM2 on downstream receptor signalling and its role in microglia function is not fully understood. Several actions were proposed for sTREM2. The sTREM2 can prompt inflammatory activation of microglia through the nuclear factor-κB. This consequently led to morphological activation and the release of pro-inflammatory cytokines.^47^ Also, sTREM2 can simulate the microglia migration and phagocytosis.^48^ Moreover, injection of sTREM2 in the brain of mice expressing amyloid precursor protein (APP) increased microglial phagocytosis of Aβ via activation and proliferation of microglia, and increased secretion of pro-inflammatory cytokines.^48^ Although the exact mechanism is unclear, it hypothesizes that the sTREM2 activates microglia. Therefore, it is crucial to understand the involvement of microglia and sTREM2 in the molecular progression of tau pathology, which encompasses tau hyperphosphorylation, elevated levels of soluble p-tau, and the emergence and propagation of fibrillary tau pathology. This is necessary to evaluate the potential of microglia and specifically TREM2-related pathways as a target for treatment.

In this study, only the subjects with available longitudinal tau-PET were included that drastically reduced the sample size. Furthermore, it was unclear whether the Aβ-negative participants developed Aβ pathology or not due to the lack of longitudinal Aβ-PET. Also, there were no AD patients in the study due to the low number at the selection step. Therefore, the current study cannot disentangle whether sTREM2 affects tau-PET in advanced Alzheimer's disease. Future studies investigating whether sTREM2 can reduce tau accumulation and spreading in advanced stages of the disease are necessary. A strength of this study is using fMRI connectome to investigate the tau spreading through functional networks that were previously observed in several studies.^29,34^ This enables us to make a more precise conclusion on the exact role of microglial activation in tau pathology.

## Conclusion

The results of this study indicate that the effect of TREM2-related microglial responses on tau pathology is stage-dependent. In addition to the previously documented positive effects of a TREM2-related microglial response in advanced Alzheimer's disease, my study demonstrates that in individuals with initial Aβ abnormalities, TREM2-related microglial activation associated is linked to reduced regional accumulation of tau aggregates and also, spreading across inter-connected brain regions, as evaluated through fMRI connectomes during the early Alzheimer's disease pathological events. These findings suggest that targeting the loss of TREM2-related microglial responses may have opposing effects on the progression of Alzheimer's disease pathophysiology when Aβ abnormalities occurred.

## Supplementary Material

fcad286_Supplementary_Data

## Data Availability

The datasets generated and/or analysed during the current study are available in the ADNI repository, https://adni.loni.usc.edu/. The datasets used and/or analysed during the current study are available from the corresponding author upon reasonable request.
